# Control Strategy Scenarios for the Alien Lionfish *Pterois volitans* in Chinchorro Bank (Mexican Caribbean): Based on Semi-Quantitative Loop Analysis

**DOI:** 10.1371/journal.pone.0130261

**Published:** 2015-06-26

**Authors:** Marco Ortiz, Fabián Rodriguez-Zaragoza, Brenda Hermosillo-Nuñez, Ferenc Jordán

**Affiliations:** 1 Instituto Antofagasta, Instituto de Investigaciones Oceanológicas, Facultad de Recursos del Mar, Universidad de Antofagasta, Antofagasta, Chile; 2 Laboratorio de Ecosistemas Marinos y Acuicultura, Departamento de Ecología, Centro Universitario de Ciencias Biológicas y Agropecuarias, Universidad de Guadalajara, Carretera a Nogales Km. 15,5, Las Agujas Nextipac, Zapopan C.P. 45110, Jalisco, México; 3 Programa de Doctorado en Ciencias Aplicadas, Mención Sistemas Marinos Costeros, Facultad de Recursos del Mar, Universidad de Antofagasta, Antofagasta, Chile; 4 Centre for Ecological Research, Hungarian Academy of Sciences, Karolina ut 29, Budapest, Hungary; 5 The Microsoft Research–COSBI, Piazza Manifattura 1, Rovereto, Italy; Dauphin Island Sea Lab, UNITED STATES

## Abstract

Ecological and eco-social network models were constructed with different levels of complexity in order to represent and evaluate management strategies for controlling the alien species *Pterois volitans* in Chinchorro bank (Mexican Caribbean). Levins´s loop analysis was used as a methodological framework for assessing the local stability (considered as a component of sustainability) of the modeled management interventions represented by various scenarios. The results provided by models of different complexity (models 1 through 4) showed that a reduction of coral species cover would drive the system to unstable states. In the absence of the alien lionfish, the simultaneous fishing of large benthic epifaunal species, adult herbivorous fish and adult carnivorous fish could be sustainable only if the coral species present high levels of cover (models 2 and 3). Once the lionfish is added to the simulations (models 4 and 5), the analysis suggests that although the exploitation or removal of lionfish from shallow waters may be locally stable, it remains necessary to implement additional and concurrent human interventions that increase the holistic sustainability of the control strategy. The supplementary interventions would require the implementation of programs for: (1) the restoration of corals for increasing their cover, (2) the exploitation or removal of lionfish from deeper waters (decreasing the chance of source/sink meta-population dynamics) and (3) the implementation of bans and re-stocking programs for carnivorous fishes (such as grouper) that increase the predation and competition pressure on lionfish (i.e. biological control). An effective control management for the alien lionfish at Chinchorro bank should not be optimized for a single action plan: instead, we should investigate the concurrent implementation of multiple strategies.

## Introduction

In the last three decades, there has been a growing interest in the study of the wide and rapid spread of the two alien lionfish species, *Pterois volitans* and *Pterois miles*, into the western Atlantic, Caribbean and Gulf of Mexico [1]. These are the first marine fish known to invade such large ecosystems [2]. The presence of this species seems to be the consequence of accidental escape or intentional introduction from aquaria in Florida in the last decade [3]. *P*. *volitans* is presently one of the most important predators in such ecosystems, reaching densities several orders of magnitude higher than observed in native environments [1]. Reports show that along the coast of North Carolina this alien species reaches an average density of 21 individuals ha^-1^ [4], and estimates from the Bahamian coral reefs reaches a mean density of 390 individuals ha^-1^ [5], which is nearly five times greater than have been reported from its native Pacific range [6].

For these reasons, the presence of this voracious, carnivorous fish could be considered as an additional perturbation factor to ecosystems already highly stressed by overexploitation, tourism, pollution, carbonate production decline and climate change [7]. Its presence could easily lead to a phase-shift transition from corals to fleshy macroalgae (as the dominant species) [8]. In addition to these disturbances, lionfish predation also decreases the overall biodiversity of coral reefs, as it consumes a high variety of invertebrate and vertebrate prey species [9, 10, 11]. Indirectly, this alien species favors the live coral cover loss because it also consumes herbivores, which reduces the grazing on algae, contributing to a shift to algal dominance [8]. Likewise, lionfish invasion is a potential human health risk due to its venomous fin spines [1]. These problems could produce negative impacts on fishery yields due to the predation and competition of lionfish on the early history stages of targeted fish, which would reduce their recruitment [12]. Likewise, the attractiveness of scuba diving destinations on Caribbean reefs will be threatened due to scenic beauty loss, generated by extensive algal overgrowth on coral [13].

One of the most relevant characteristics of species introduced into a non-natural ecological system is to show *r*-type dynamics [14]. The lionfish *P*. *volitans* is a clear example because of its ability to spread in different environments, ranging from the outer margins of reefs to nursery habitats such as sheltered mangrove lagoons [15]. In the Atlantic, lionfish shows high individual growth and reproductive rates [13] and a high population growth rate [8]. This behavior could be explained by at least the following two reasons: (1) lionfish present predatory features that are not recognized by the other species in invaded habitats, so that their prey cannot detect it as a potential threat, making it a more successful predator than native predators (i.e. native local community) [9]. This fish thus exhibits high predation efficiency (as an ambush-unknown predator) mainly upon reef-fish species, including economically important fish and crustaceans [16]; (2) this species shows a reduced mortality by predation, as most of its putative predators are present only in low densities as a consequence of intensive (historical) exploitation [17], even reaching larger sizes in invaded habitats compared to those recorded in its native ecosystems [9]. Under such a particular scenario, the receiving ecological system could be dominated by positive feedbacks at different levels of complexity (within the network), showing unstable states [18]. For this reason, there is a growing concern that lionfish will affect the structure and function of invaded marine ecosystems [1].

The study of a species within ecological and management context encounters at least two types of constraints. On the one hand, the ecological system we wish to manage is composed of a network of interacting populations, therefore, any natural or human intervention percolates through this network, being amplified along some pathways, buffered along others, and possibly even inverted. On the other hand, our interventions are frequently not constant: we act on the system and also respond to it, so that our actions co-vary with the variables of the natural system, therefore we may introduce uncertainties [19]. Different research strategies, which are not mutually exclusive, can be used to study, assess, and attempt to predict the transformations in a natural system as a response to human interventions and/or introduction of exotic species. These strategies include (1) the reduction of objects of study to their small parts, assuming that this subsystem represents well the whole original ecosystem; (2) the statistical analysis of factors (weighted by their relative importance); (3) quantitative simulation requiring fairly precise measurements of the variables and parameters and exact equations (this is quite difficult for variables that cannot be measured); and (4) semi-quantitative or qualitative models (*Loop Analysis*) that do not need precise nor quantitative equations: these allow the integration of non-measurable variables and physically different forms, focusing on the nature of the change (e.g. its sign) rather than its precise magnitude [20]. Likewise, it is a useful technique for estimating the local stability (as a component of sustainability) of systems [20] and assessing the propagation of direct and indirect effects as a response to external perturbations [21]. This approach has been applied widely in different fields of the natural sciences [19, 22, 23, 24,].

Due to the difficulty in carrying out replicated experiments to estimate stability properties under different regimes of disturbances (such as fishing and pollution) [25, 26], we chose loop models to capture the general ecological and eco-social interactions underlying the invasion and spread of the lionfish *P*. *volitans* in the Chinchorro bank coral reef ecosystem (Mexican Caribbean). Simultaneously, we assessed the local stability (as an approach to holistic sustainability) of a set of different ecological and eco-social models in response to alternative control management scenarios to reduce the abundance of this alien species. We note that our models should be considered as complementary and extended versions of the recently published age-structured population model for the Western Atlantic Ocean [27] and quantitative trophic models for the Caribbean coral reef ecosystem) [28] developed for lionfish recently.

## Material and Methods

### Ethic Statement

No protected and endangered species were involved in this study. No vertebrate neither invertebrate species were collected in the present work. No sampling program was performed *in situ* because the models were built taken information from scientific literature. No specific permissions were required for this study area neither for the intellectual work.

### Study area

For this work, we modeled the ecological coral reef system of Chinchorro bank (18° 35’ N; 87° 23’ W), Mexican Caribbean, which is located 30.8 km to the southwest of the Yucatán Peninsula. It is separated from the continent by a channel nearly 500 m in depth [29]. Chinchorro bank is of ovoid shape, 43.2 km long and 18.0 km wide [30]. The reef lagoon is surrounded by a semi-continuous barrier reef with a perimeter of approximately 115 km, an area exceeding 500 km^2^, and depths between 1 and 9 m in the south, and 2 m in the north. The reef patches and coral knolls decrease in number and size from south to north [31].

Chinchorro bank is one of the largest platform coral reefs of the Caribbean Sea [29] and was declared as a Biosphere Reserve in 1996 by the Mexican government [28]. Chinchorro bank has received little impact from tourism and coastal human populations due to its isolation. Nevertheless, it has been subjected to intense fishing activities for over 40 years, mainly targeting *Panulirus argu*s (Caribbean spiny lobster), *Strombus gigas* (queen conch), and various other fish species. These fisheries have strongly impacted *P*. *argus* and *S*. *gigas*, decreasing their abundances since the late 1980s. The exploitation of reef fishes belonging to the families Serranidae (groupers), Lutjanidae (snappers), and Haemulidae (grunts) in Chinchorro Bank has also been permanent and historical. However, the reef fish harvest increased especially when the Mexican fishing authority (dependent on the federal government) implemented bans for the harvest on the queen conch (*S*. *gigas*) and the spiny lobster (*P*. *argus*). Likewise, the reef of Chincorro Bank includes four cays that cover 0.4% of the total reserve surface, named North Cay at the north, Central Cay in the center-east of the system, and Lobos Cay in the south. The seawater is oligotrophic with sea surface temperature ranging from 27 to 29° C, and salinity ranging from 36.6 to 36.9 ‰ [30]. Trade winds dominate this coral reef across the year, although northern winds predominate between October and May [31]. In summer, the reef is exposed to tropical storms and hurricanes that can reach level 5 on the Saffir-Simpson scale, as was the case of Hurricane Dean in 2007.

### Loop Analysis, a semi-quantitative modeling approach

**Fig 1:**
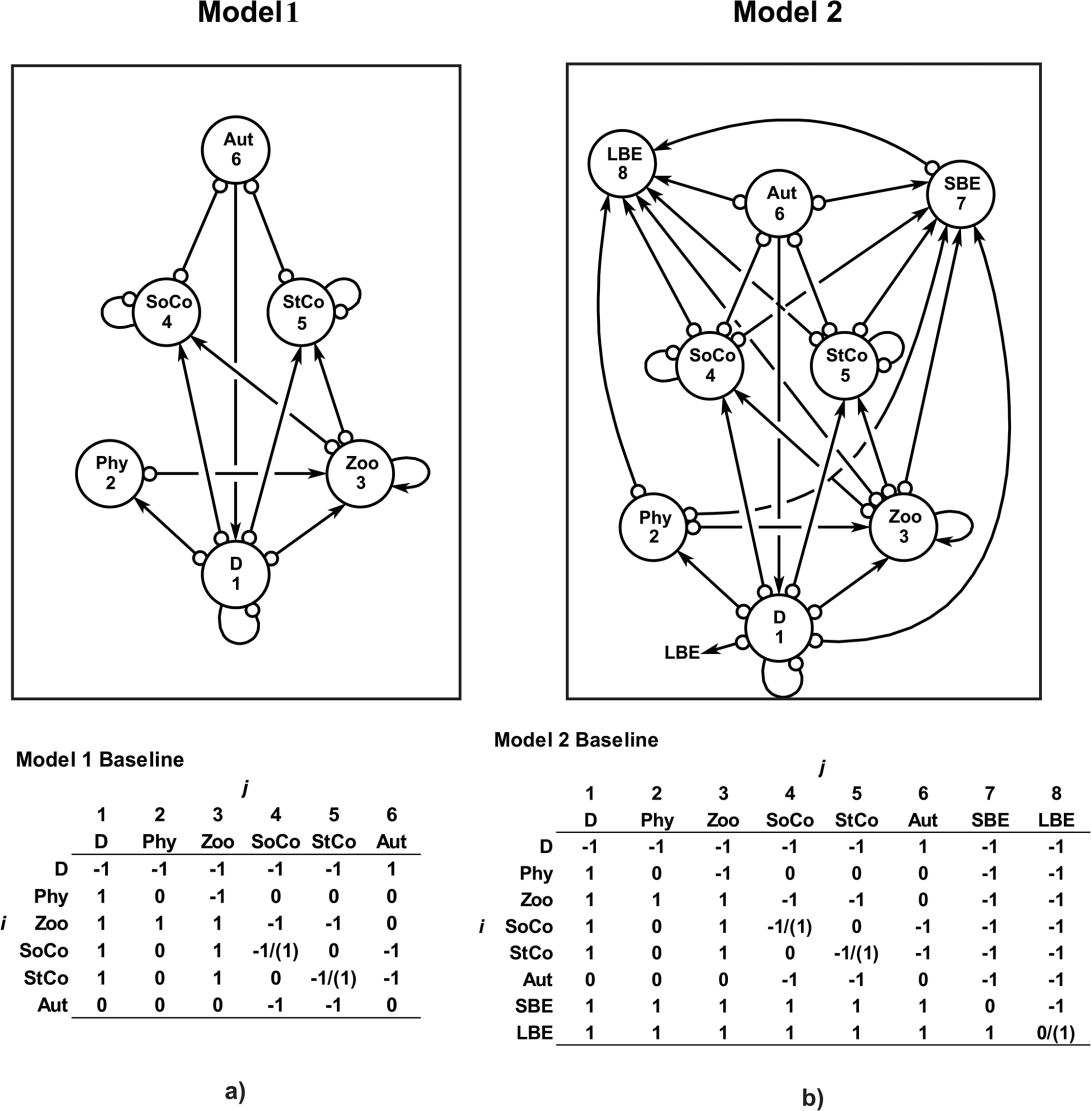
Models 1 and 2. Ecological models 1 and 2 for the coral benthic system of Chinchorro bank (México). The baseline community matrices with the nominal effect of *j* variable to *i* are also shown. The parenthesis shows the kind of intervention. For more explanation of the name of variables and interactions see the text.

Models of populations, communities or ecosystems represent only some selected relations of the studied ecosystems in a qualitative or quantitative way [20]. Loop models provide a qualitative or semi-quantitative framework for formulating the relationships between variables within a particular system. It is also possible to estimate the local stability properties of the system (sustainability) and to determinate the effects of external factors on the variables [20]. Loop models show only the sign of a relationship, which indicates the type of influence each variable has upon another (i.e., positive, negative or zero) (Fig 1). In ecological interactions, (+,-) denotes a predator-prey, parasite-host, or resource-consumer relationship, (-,-) represents competition between two species, whereas (+,+), (+,0), and (-,0) represent mutualism, commensalism and amensalism, respectively. Each variable is represented by a node (large circle) and edges (lines) representing directions and types of relationships: an arrow at one end indicates a positive effect, a circle means the effect is negative and the lack of a symbol shows a null effect. *Loop Analysis* is based on the correspondence between systems of differential equations at equilibrium, community matrices and loop diagrams. Therefore, in the system, the element *a*_*ij*_ of the matrix and the loop diagram represent the effect of variable *j* on the growth of variable *i* when the equation:

$$\frac{dX_{i}}{dt}=f_{i}\left(X_{1},X_{2},X_{3},\ldots ,X_{n};C_{1},C_{2},C_{3},\ldots ,C_{n}\right)$$ (1)

where the change in time of variable *X*_*i*_, is a function *f*_*i*_ of other interconnected variables *X*_*n*_ and parameters *C*_*n*_ and is solved at equilibrium (X*). The link from *X*_*j*_ to *X*_*i*_ is similar to the *α*_*ij*_ in [32], as follows:

$$\alpha_{ij}=\frac{\partial \left(\frac{dX_{i}}{dt}\right)}{\partial X_{j}}$$ (2)

where *X*_*i*_ is evaluated at a moving equilibrium for the system. The element of the graph representing the link from *j* to *i* is sign (*α*_*ij*_)–whether positive, negative, or zero- where the function sign (*X*) is 1 when *X* > 0, 0 when *X* = 0, and -1 when *X* < 0.

Local stability, as determined by the Routh-Hurwitz criteria, translates into loop terms as Condition 1 when *F*_*k*_ < 0 for all *k*; i.e., *F*_*k*_ corresponds to the negative feedback on every level of complexity (*k*) that must exceed the positive feedback. Condition 2 examines the relations among feedbacks at different level *k*, using Routh-Hurwitz inequalities. This criterion indicates that a moving equilibrium could be asymptotic or damped oscillatory stable, or contrary divergent or undamped oscillatory unstable. This second condition was calculated by using the expansion of the Routh-Hurwitz determinants in terms of feedbacks or loops. In the case of a system of three variables the first inequality corresponds to *F*_*1*_ *F_*2*_ *+F*_*3*_ *> 0* [18, 20]. The feedback for each level can also be calculated by estimating the characteristic polynomial related to the *Jacobian* interaction matrix, in which the polynomial now can be written in terms of the feedback notation as: *F*_*0*_ *λ* ^*n*^ *+F*_*1*_ *λ* ^*n-1*^ *+F*_*2*_ *λ* ^*n-2*^ *+…*..*+F*_*n-1*_ *λ + F*_*n*_ = 0, where *F*_*0*_ ≡ -1 and the *F*_*n*_ is the feedback of the entire system (*n* = total number of variables in the system). The Levins’s stability criterion assumes that the system is locally stable when *F*_*n*_ is negative. The stronger the negative feedback (*F*_*n*_) becomes, the greater the resistance will be to external change [20]. Based on this local stability criterion, it is possible to estimate the degree of resistance to perturbations (as a measure of sustainability) of the system and, simultaneously, to explore strategies to increase this resistance. Likewise, the semi-quantitative loop models allow indicate what must be measured by identifying the self-dynamics and interactions that changes the local stability [20]. It is relevant to indicate that the *Loop Analysis* does not permit to assess the effect of the extinction of some variable on the model properties. In the present study, this framework helps us to determine what scenarios for reduction of lionfish density (as control mechanism) are locally stable (as an approach to holistically sustainable).

### Selection of network boundaries, structure and assumptions

Five semi quantitative models were constructed considering the most important variables and interactions before and after the invasion of the lionfish *P*. *volitans*. In the simplest version (model 1), six variables are represented, and sequential models expand the boundaries until the eco-social version of the model (model 5) contains 19 variables. It is important to indicate that models 1, 2 and 3 represent different levels of ecological complexity before the invasion of lionfish, whereas in models 4 and 5 we added lionfish and social variables.

Most of trophic relationships among the variables were taken from [28], It should be noted that, in all the models, some variables (the most relevant ones) were considered self-damping (density-dependent growth rates) due to higher density close to carrying capacity or self-enhanced (density-independent growth rates) as response to intensive harvest (reduction of abundance) [19].Both self-dynamics can be demonstrated using the following difference (Ricker’s) equation:

$$f(x)=x\cdot {\text{exp}}^{r\left(1-\frac{x}{k}\right)}$$ (3)

where *x* is the abundance of a variable (species or functional group), *r* = intrinsic growth rate and *k* = carrying capacity. This difference equation is considered the analogue of the logistic differential equation with similar parameters.

For restoration or re-stocking program of a variable (self-damped dynamics), consider the following extension of Eq 3:

$$f(x)=x\cdot {\text{exp}}^{r\left(1-\frac{x}{k}\right)}+\frac{b}{x},$$ (4)

where *b/x* = the restoration or re-stocking rate of *x*. The derivative of (4) with respects to *x* (self-damped dynamics) becomes as:

$$\frac{\partial f(x)}{\partial x}={\text{exp}}^{r\left(1-\frac{x}{k}\right)}-\frac{x\cdot r}{k}{\text{exp}}^{r\left(1-\frac{x}{k}\right)}-\frac{b}{x^{2}}$$ (5)

therefore, the restoration or re-stocking of *x* will cause self-damping on itself.

By another side, the over-exploitation or destruction of *x* (self-enhanced dynamics) becomes as:

$$f(x)=x\cdot {\text{exp}}^{r\left(1-\frac{x}{k}-\frac{h}{x}\right)},$$ (6)

where the parameter *h/x* is the harvest (or destruction) rate of *x*. The derivative of Eq (6) with respects to *x* (self-dynamics) becomes:

$$\frac{\partial f(x)}{\partial x}={\text{exp}}^{r\left(1-\frac{x}{k}-\frac{h}{x}\right)}+\frac{x\cdot r\cdot h}{x^{2}}{\text{exp}}^{r\left(1-\frac{x}{k}-\frac{h}{x}\right)}-\frac{x\cdot r}{k}{\text{exp}}^{r\left(1-\frac{x}{k}-\frac{h}{x}\right)}$$ (7)

thus, the over-exploitation or destruction of *x* will cause self-enhanced dynamics on *x*.

#### Model 1

This model includes six variables: detritus (D) (as a complex of microorganisms and nutrients), phytoplankton (Phy), zooplankton (Zoo), soft corals (SoCo) (constituted by all octocoral, such as *Pseudopterogorgia* spp., *Muricea* spp., *Plexaura* spp., *Pterogorgia* spp., and others), stony corals (StCo) (formed by hermatypic corals [*e*.*g*., *Montastraea* spp., *Diploria* spp., *Siderastrea* spp., *Porites* spp., *Agaricia* spp., and others] and hydrocorals [*i*.*e*., *Millepora* spp.]), and a group of benthic autotrophs (Aut) (including fleshy macroalgae, crustose calcareous algae, articulated calcareous algae, and seagrass). Most of these groups were connected by prey-predator relationships. An exception was the Aut, with a positive impact to detritus, and the negative interaction (competition) between SoCo and StCo with the Aut. No interaction between SoCo and StCo was considered because rigorous information is limited. The D, SoCo and StCo were self-damped, that is, their abundance close to carrying capacity (healthy conditions), and Zoo is self-enhanced due to high mortality and low density by predation (Fig 1A). Table 1A shows the scenarios considered for the sustainability (stability) assessment.

**Table 1:** Routh-Hurwitz and Levins´s stability criteria (as sustainability measure) for the different models and scenarios simulated.

		Stability criteria		
		Routh-Hurwitz		Levins
Model/Intervention	Assumptions	1°C	2°C	Fn
(a) Model 1				
Baseline	SoCo-, StCo-, Aut0	yes	no	(-)4
Scenario 1A	SoCo+, StCo+, Aut0	no	no	(+)4
(b) Model 2				
Baseline	SoCo-, StCo-, Aut0, LBE0	yes	yes	(-)4
Scenario 2A	SoCo+, StCo+, Aut0, LBE0	no	yes	(+)4
Scenario 2B	SoCo-, StCo-, Aut0, LBE+	no	yes	0
(c) Model 3				
Baseline	SoCo-, StCo-, Aut0, LBE0, HFa0, CFa0	no	yes	(-)4
Scenario 3A	SoCo+, StCo+, Aut0, LBE0, HFa0, CFa0	no	yes	(+)6
Scenario 3B	SoCo-, StCo-, Aut0, LBE+, HFa+, CFa+	no	yes	(-)32
Scenario 3C	SoCo+, StCo+, Aut0, LBE+, HFa+, CFa+	no	yes	(+)56
(d) Model 4				
Baseline	SoCo-, StCo-, Aut0, LBE0, HFa0, CFa0, LF+	no	yes	(-)6
Scenario 4A	SoCo+, StCo+, Aut0, LBE0, HFa0, CFa0, LF+	no	no	(+)12
Scenario 4B	SoCo+, StCo+, Aut0, LBE+, HFa+, CFa+, LF+	no	no	(+)220
Scenario 4C	SoCo-, StCo-, Aut0, LBE+, HFa+, CFa+, LF+	no	no	(-)132
Scenario 4D	SoCo+, StCo+, Aut0, LBE+, HFa+, CFa-, LF+	no	no	(+)356
Scenario 4E	SoCo-, StCo-, Aut0, LBE+, HFa+, CFa-, LF+	no	yes	(-)228
(e) Model 5				
Baseline	F1 exploits LBE, HFa, CFa; and F2 exploits only LFs	no	yes	(+)33
Scenario 5A	F1 exploits LBE, HFa, CFa, LFs; and F2 exploits only LFs	no	yes	(-)15
Scenario 5B	F1 exploits LBE, HFa, CFa, LFs; and F2 exploits LFs, LFd	no	yes	(-)20
Scenario 5C	F1 exploits LBE, HFa, CFa, LFs, LFd;and F2 exploits LFs, LFd	no	yes	(+)3
Scenario 5D	F1 exploits LBE, HFa, CFa, LFs, LFd;and F2 exploits LFs, LFd and increases Cfa	no	yes	(-)2
Scenario 5E	F1 exploits LBE, HFa, LFs, LFd;and F2 exploits LFs, LFd	no	yes	(+)16
Scenario 5F	F1 exploits LBE, HFa, LFs, LFd;and F2 exploits LFs, LFd and increases CFa	no	yes	(+)52

Local stability measures Routh-Hurwitz and Levins (*F*_*n*_) criteria in the models and scenarios simulated. First criterion (1°C) describes stability condition, and the second criterion (2°C) determines asymptotic or oscillation condition. The Levins’s (*F*_*n*_) criterion can be used as an approach for holistic sustainability. The assumptions considered were changes in the self-dynamics (damped ´-´and/or enhanced ´+´) for the variables in each model^aa^ The names of the variables are described with details in Methods section.

#### Model 2

This model is the extension of model 1, including two new variables: the small benthic epifauna (SBE) and the large benthic epifauna (LBE). The SBE comprises all small species that live on reef benthos as amphipods, bivalves, chitons and gastropods, crabs, shrimps, barnacles, bryozoans, hemichordates, isopods, polychaetes, tunicates, and other organisms. The LBE incorporated larger species as asteroids, holothurians, echinoids, ophiuroids, octopuses, large gastropods (*e*.*g*., *S*. *gigas*), lobsters (*e*.*g*., *P*. *argus*), large crabs, sponges, and others. Both variables were connected by predation with the other groups (Fig 1B). Table 1B summarizes the scenarios simulated for the sustainability estimates.

#### Model 3

**Fig 2:**
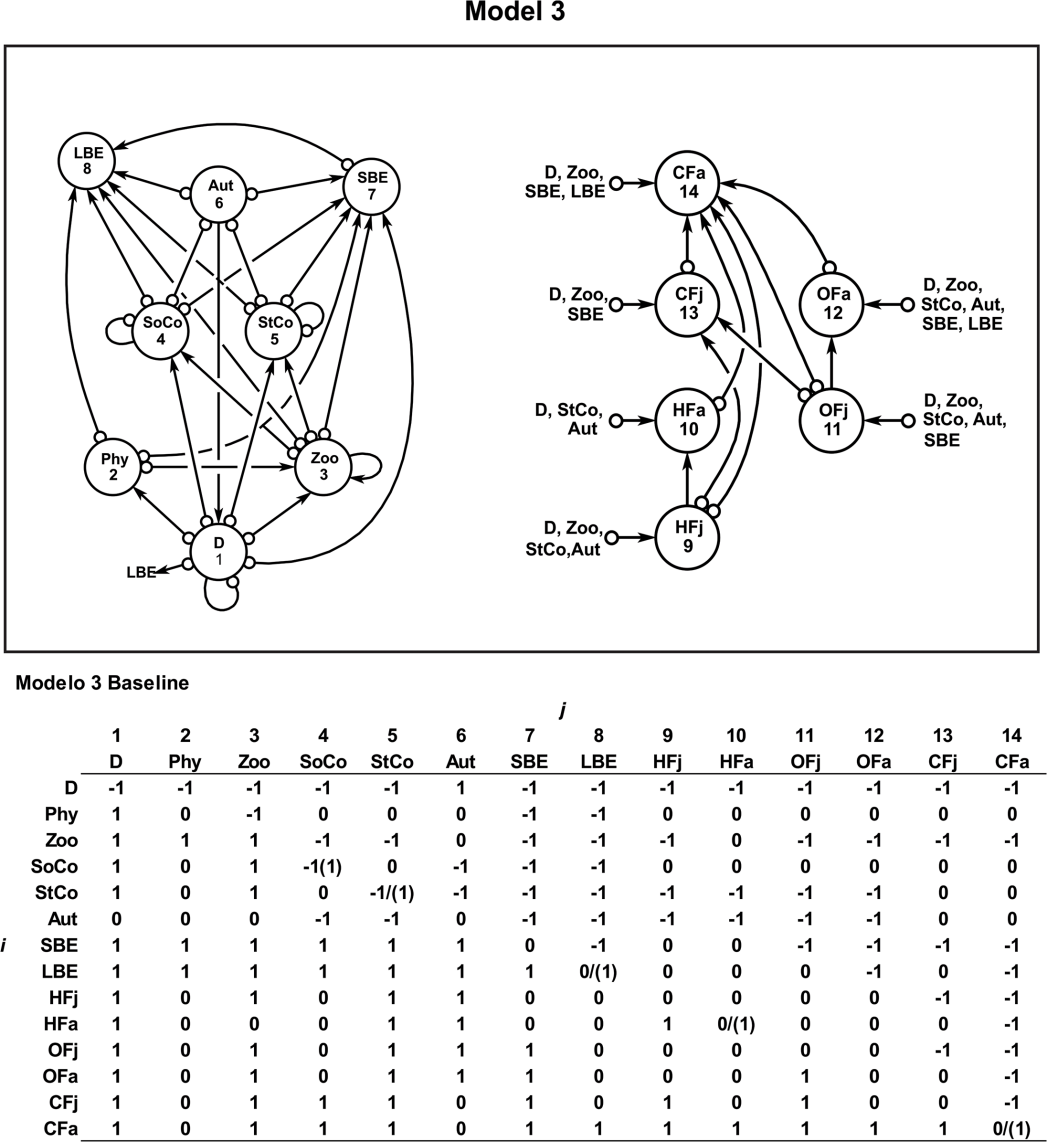
Model 3. Ecological model 3 for the benthic-pelagic coral system of the Chinchorro bank (México). The baseline community matrix with the semi-quantitative effect of *j* variable to *i* variable is also shown. The parenthesis shows the kind of intervention. For details see section of Methods.

This model includes yet another six variables: (1) herbivore fish juveniles (HFj) and adults (HFa) principally corresponding to scarids (parrotfishes), acanthurids (surgeonfish); (2) omnivore fish juveniles (OFj) and adults (OFa), which were mainly accounted for by damselfish; and (3) carnivore fish juveniles (CFj) and adults (CFa), representing groupers, snapper, jacks, barracudas, grunts and other reef carnivore fish. Within this model HFj and OFj have a positive influence on HFa and OFa, respectively. These relationships are similar to those described in population models [16]. CFj and CFa were connected by predation due to cannibalism (Fig 2). The CFa was considered as top predator, which preys on OFa, OFj, HFa, and HFj. The HFj and HFa have a negative (direct) influence on StCo as a consequence of an incidental depredation.The scenarios considered to evaluate stability are visualized in Table 1C.

#### Model 4

**Fig 3:**
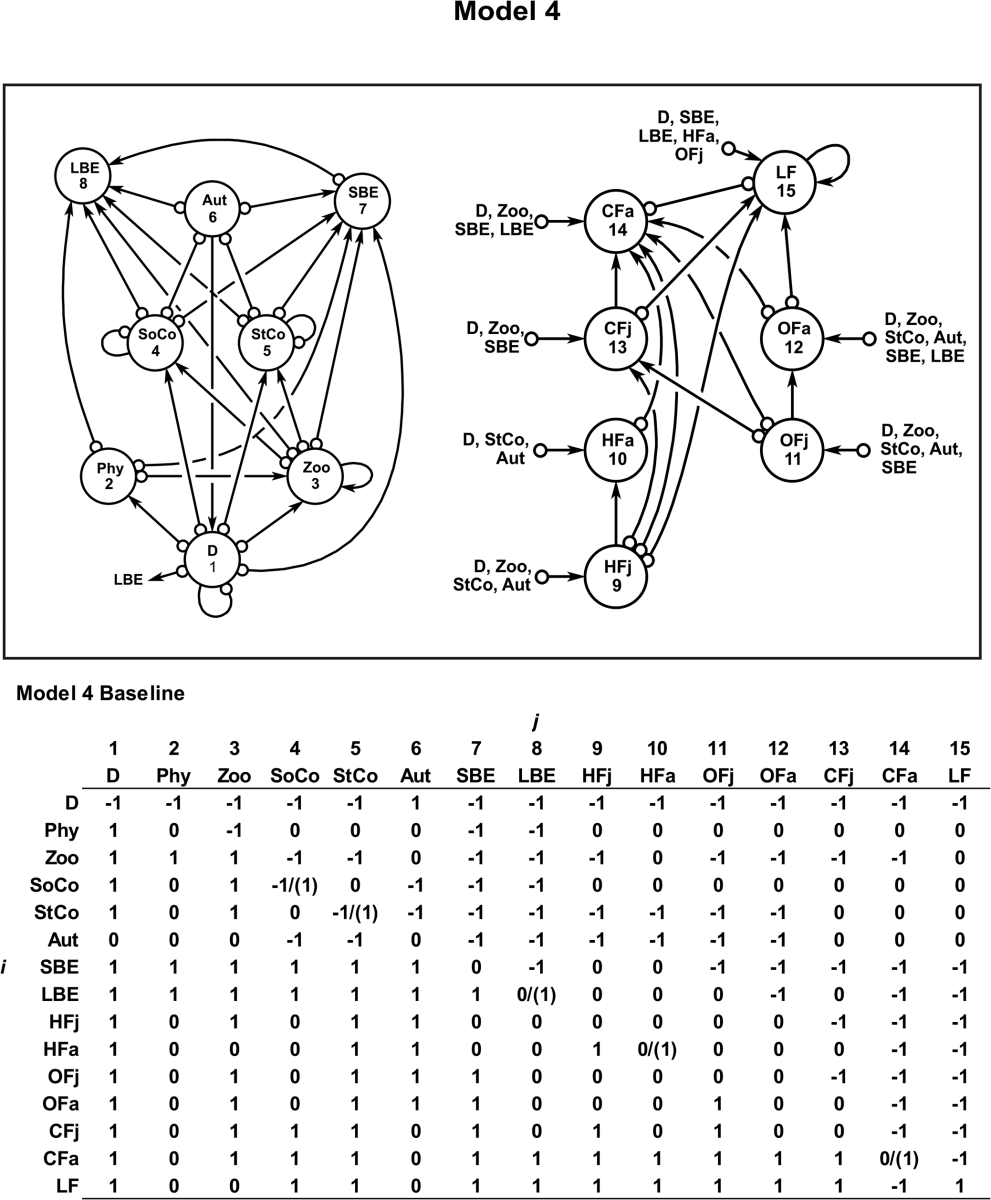
Model 4. Benthic-pelagic ecological model 4, including the alien lionfish (LF) into the benthic-pelagic system of the Chinchorro bank (México). The baseline community matrix with the semi-quantitative effect of *j* variable to *i* variable is also shown. The parenthesis shows the kind of intervention. See Methods for more details.

This model is the extension of model 3, including also the alien lionfish *P*. *volitans* (LF). This variable was connected as predator upon D, SBE, LBE, HFj, HFa, OFj, OFa, and CFj. The lionfish and CFa were qualitatively simulated as competitors due to cross-predation (Fig 3). The scenarios considered for the assessment of sustainability are described in Table 1D.

#### Model 5

**Fig 4:**
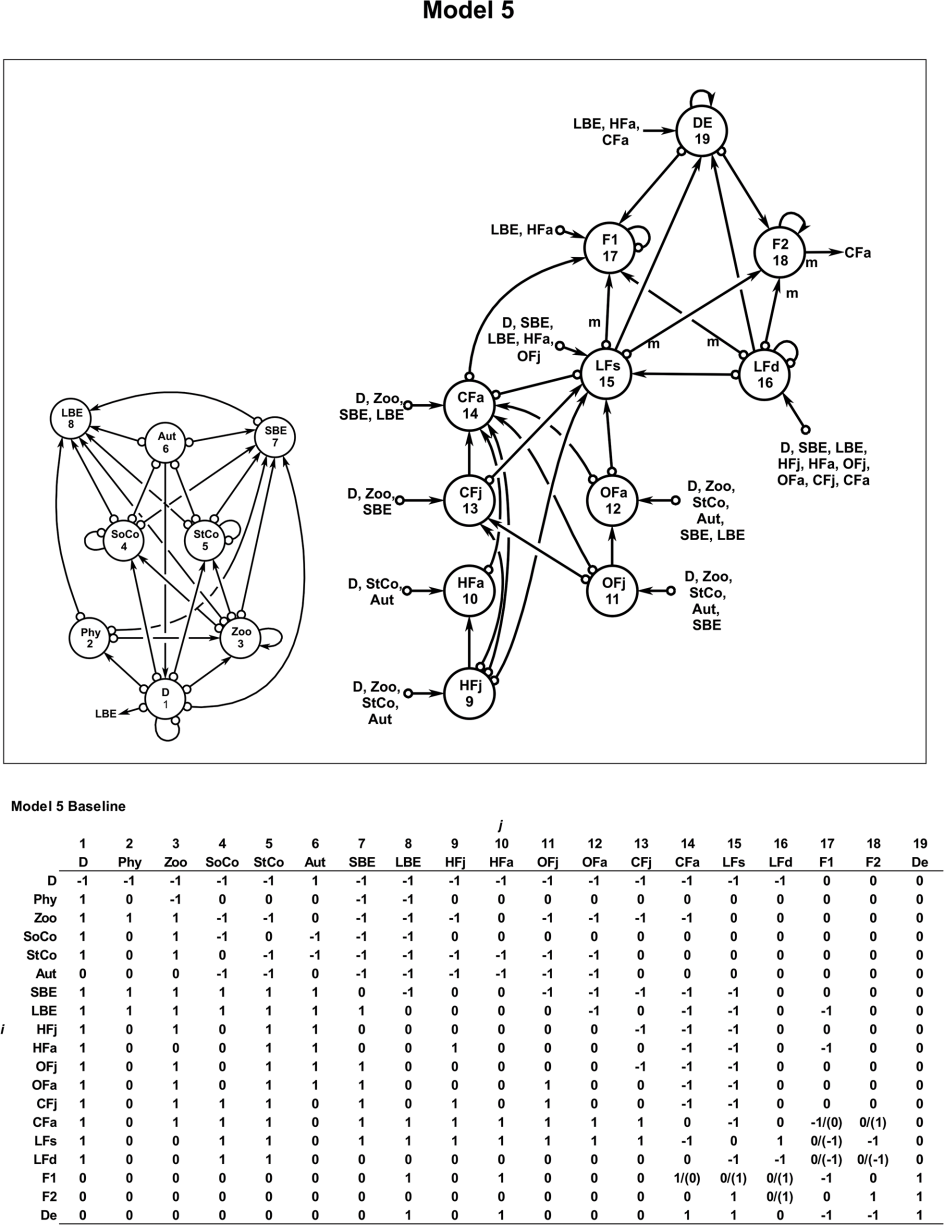
Model 5. Ecological and social model 5 for the Chinchorro bank (México). The lionfish is separated in two meta-populations (from shallow and deeper waters) and two kinds of fishers and the demand (from the market) are also integrated. The baseline community matrix with the semi-quantitative effect of *j* variable to *i* variable is also shown. The parenthesis shows the kind of intervention. For more details of the variables and interactions see the text (Methods).

Finally, this model is a modification of model 4, separating the *P*. *volitans* into two variables; the lionfish from shallow waters (LFs), corresponding to organisms inhabiting benthic systems < 40 m depth, and the lionfish from deeper waters (LFd) inhabiting benthic systems > 40 m depth. Both variables were related to the remaining ecological variables in a manner similar to LF in model 4. In this model, we assume LFd is self-damped (density-dependent growth rate) as a consequence of this group not being exploited (near carrying capacity) and that LFd migrates to shallow water, increasing the abundance of LFs. Likewise, two variables related to fisheries were included: artisanal fishermen (F1) exploiting LBE, HFa, CFa, and LFs, and control fishermen (F2) harvesting exclusively on LFs. The F1 were limited (self-damping) in the number of boats permitted, and their harvest activities are controlled (by legislation), whereas the F2 were self-enhancing because their activity is promoted by the authority. F1 and F2 were connected to LFs and other groups by predation. The demand (DE) was also included, and it has a positive influence on fishermen (F1 and F2), and the fishermen have a negative effect on demand. This relationship represented a situation where the prices would be determined by local market [19]. All commercial groups stimulate demand positively. DE is self-enhancing because the market is dominated by positive feedbacks [33] (Fig 4). Table 1E shows the scenarios simulated for the sustainability estimate. Due to the high complexity represented by this model, the soft corals (SoCo) and stony corals (StCo) were only simulated under self-damping dynamics, which would be a consequence of a permanent restoration program.

## Results

Model 1 shows that a perturbation impacting stony and soft-corals by reducing their abundances far below their carrying capacity would produce an unstable (unsustainable) ecological system under Routh-Hurwitz and Levins’s criteria (Table 1A). On the other hand, any restoration program (baseline model) for corals that increases their abundances (near carrying capacity) would be stable or sustainable (Table 1A) (Fig 1A). In the case of model 2 (including two new variables) (Fig 1B), a similar pattern to model 1 is observed regarding the changes in coral cover (Table 1B); however, an intensive exploitation of large benthic epifaunal species (LBE) was found only to be asymptotically locally stable (scenario 2B) (Table 1B).

Table 1C presents the stability measurements from model 3 (Fig 2). In the case of the restoration program for corals, stability would be partially obtained (baseline model). It is important to indicate that under any restoration program for corals and an exploitation of LBE, HFa and CFa would also be partially sustainable (baseline model) (Table 1C). However, if any perturbation impacts stony and soft-corals negatively and simultaneously the fishermen intensively exploit LBE, HFa and CFa induced dynamics that produce an unsustainable (unstable) system (Table 1C).

The stability outcomes obtained from model 4 (Fig 3), which integrates the lionfish (LF) as a variable into the system, are summarized in Table 1D. It is relevant to note that the most sustainable scenario was obtained under the following simultaneous conditions: (1) restoration program for corals, (2) fishing on LBE, HFa and LF, and (3) implementation of bans for the fishery of carnivore fishes (CFa) (scenario 4E) (Table 1D). Likewise, any negative perturbation on corals would drive the system towards unstable (unsustainable) states, which agree with the results obtained in the model 1, 2 and 3 (Table 1A, 1B, 1C and 1D).

Table 1E shows the results of stability for the eco-social model 5 (Fig 4). In this case the system reached the most highly partially stable state when the F1 exploits LBE, HFa, CFa and LFs, and F2 exploits LFs and LFd (scenario 5B) (Table 1E). Therefore, an exploitation program of lionfish inhabiting deeper waters should also be implemented. Likewise, a re-stocking program to increase the abundance of the carnivorous fish (CFa) (as grouper species) would also achieve the necessary stability (scenario 5D) (Table 1E). All characteristic polynomials regarded each model/scenarios are summarized in S1 Text.

## Discussion

It is widely recognized that accidental or planned introductions of alien species primarily motivated by commercial and recreational purposes have resulted in significant ecological disturbances in aquatic and terrestrial systems, promoting the local reduction of native populations (in some cases to the point of extinction, resulting in biodiversity loss) [1].

The qualitative or semi-quantitative models built and analyzed herein correspond to a partial representation of the variables and interrelationships underlying the ecological and eco-social dynamics of the Chinchorro bank ecosystem. This caveat, however, is applicable to any type of model and independent of its level of complexity [34, 35]). In this sense, we recognize that the models presented have made use of at least five sources of simplifications: (1) we reduced the system complexity through the composition of functional groups for the most variables of the model, an exception was the lionfish *P*. *volitans*; (2) only the native fishes were separated in two class groups (juveniles and adults) without enough scientific information about their own dynamics, (3) we assumed the system (community-matrix) to be in a moving equilibrium, (4) we included only the fishermen and demand coming from socio-economics field, and (5) regardless of the well-known limitations of the *Loop Analysis* theoretical framework, the models constructed represent the processes underlying the systems analyzed when only considering short-term dynamics. Despite these limitations, we claim that the results obtained are sufficiently robust given the agreement among models of different levels of complexity, therefore permitting us both to compare different management strategies to control the alien lionfish and to assess their consequences in the local stability or sustainability of the Chinchorro coral ecosystem. Moreover, for our purposes, the moving equilibrium is understood to be a trajectory path in a short dynamic that includes the set of variables of the system from which the system will not move unless perturbed. *Loop Analysis* is used to determine the direction of change of the equilibrium values of any variable in response to changes in any parameter [18]. Likewise, it is recognized for its high correspondence between model predictions and observed-empirical responses [36, 37, 38, 39], which is possible based on stable qualitative community matrices.

The results obtained in the models 1, 2, 3, 4, and 5 showed clearly that a restoration program for the soft- and hard corals, that is under self-damped dynamics, emerge as a locally stable or sustainable human intervention independent of the model complexity level. This result agrees with experimental studies in which the construction of artificial coral reefs improved the health (increasing biodiversity and ecological complexity) of historically highly perturbed ecosystems (i.e., that have suffered from stresses including fisheries, pollution, and eutrophication) [40]. Models 2 and 3 (before lionfish invasion) and model 4 with *P*. *volitans* showed that the exploitation of species belonging to the LBE, HFa and CFa functional groups would be possible only if a restoration program for corals is implemented concurrently. Likewise, the harvest of lionfish achieves the highest local stability or sustainable control management only when the CFa are not exploited, agreeing with the conclusions described by [41, 42]. It is important to indicate that species of grouper (within CFa) such as *Epinephelus striatus* and *Mycteroperca tigris* feed upon lionfish [43]. It has also been described that lionfish appeared to remain closer to refuges at places with high grouper densities, suggesting that the grouper may reduce both the abundance and consumption rates of lionfish [44]. However, it is not clear whether recruitment of lionfish in non-reef benthic habitats without large predatory groupers results in an increase (by migration) of lionfish in reef systems [45].

In the case where the lionfish from shallow and deeper waters are considered separately, the situation is quite complex because the most sustainable scenario is achieved if the artisanal fishermen exploit shallow stocks of lionfish and the control fishermen simultaneously exploit both lionfish groups (scenario 5B). In contrast, if the control fishermen harvest only shallow stocks of lionfish, the system would be locally unstable (baseline). These qualitative responses do not agree with the outcomes described by [27, 28], who simulated dynamically different harvest scenarios considering only lionfish from shallow waters. Therefore, more efforts should be focused towards the implementation of new exploitation strategies, particularly for lionfish in deeper waters because this group could be a source population for local shallow-water populations. This additional control strategy would be especially necessary in coastal areas where the harvest has provoked local extinctions of large predator fish [46]. In such scenarios, a re-stocking program for CFa (as groupers) should also be considered by the Mexican authorities because this intervention would promote a holistic sustainability of the entire system (scenario 5D). In this sense, large-bodied predatory fishes could be capable of controlling the fast spread and population explosion of lionfish [4], although this opinion should be taken with caution as there is evidence that native predators would not influence invasion success (i.e., colonization or post-establishment population density) of lionfish on Caribbean reefs [47].

Our models show that an exploitation of *P*. *volitans* from shallow and deeper waters seems to be a suitable control mechanism for this species at Chinchorro bank. However, this management strategy should be pursued with care because exploitation for the human consumption market could propagate unexpected negative long-term consequences (since this species could be introduced in many other places for commercial proposes [48]. Likewise, it is necessary to consider that complex ecosystems subjected to exploitation are continuously changing (exhibiting oscillatory dynamics as a consequence of the relative dominance of positive feedbacks), which could promote negative effects on the health of the systems, reducing the effectiveness of this control mechanism for *P*. *volitans*.

## Conclusions

The loop model outcomes obtained in this work help us to better understand the possible consequences of different control strategies for *P*. *volitans* at Chinchorro bank, especially when the population dynamics of any species depends on a complex network of intercactions among the biotic and abiotic components of the ecosystem [49]. The situation is even more complex if we also consider the populations as meta-populations heterogeneously scattered in an environment, which is also heterogeneous, where the extinction and colonization rates of an exotic species would depend on the superposition of different co-existing meta-populations [50]. Although the harvest on lionfish from shallow waters as a control mechanisms was found to be locally stable, agreeing in general terms with [25, 26, 51], it remains necessary to plan other additional human interventions (within an ecosystem-wide adaptive management program), which should include: (1) the restoration of corals, (2) the exploitation of lionfish from deeper waters, (3) the implementation of bans and re-stocking program for carnivore fishes (as grouper), and (4) the exploitation of juvenile lionfishes, which in turn fed on recruits of native herbivore, carnivore and omnivore fishes [12]. Nevertheless, these management strategies should be implemented simultaneously because implementation in isolation could have a negative impact on lionfishes only at a local spatial scale [46]. Even though the current contribution shows the importance of combining different types of studies that tackle the issue from several angles, thereby providing robust conclusions, however, additional explorations should be also performed in order to assess the impact of changes in the sign of interactions on the local stability properties of the system. Therefore, we should seek to implement multiple and simultaneous strategies to reduce the exotic lionfish population at Chinchorro bank (Mexico) and bring it within sustainable boundaries. Additionally, the approach developed here should be considered a general strategy for examining the consequences of natural changes and human interventions in ecosystems.

## Supporting Information

S1 Text Characteristic polynomials. Characteristic polynomials, p(λ), for each model and scenarios simulated. The polynomials were multiplied by (-1) due to F_0_ ≡ -1 (for more details see Methods).
(DOCX)
