# Supplementary material for: Control Strategy Scenarios for the Alien Lionfish Pterois volitans in Chinchorro Bank (Mexican Caribbean): Based on Semi-Quantitative Loop Analysis
Source: PLoS One. 2015 Jun 26;10(6):e0130261. doi: 10.1371/journal.pone.0130261 (PMC4482547; doi:10.1371/journal.pone.0130261)
Supplement: S1 Text — Characteristic polynomials, p(λ), for each model and scenarios simulated. The polynomials were multiplied by (-1) due to F0 ≡ -1 (for more details see Methods). (DOCX) [file pone.0130261.s001.docx]

**Text S1**

**Characteristic polynomials.** Characteristic polynomials, p(λ), for each model and scenarios simulated. The polynomials were multiplied by (-1) due to F_0_ ≡ -1 (for more details of methodology see Text S2).

**Model 1**

Baseline: p(λ) : -λ^6^-2λ^5^-5λ^4^-8λ^3^-8λ^2^-8λ-4

Scenario 1A: p(λ): -λ^6^+2λ^5^-5λ^4^+4λ^3^-4λ^2^-λ+4

**Model 2**

Baseline: p(λ): -λ^8^-2λ^7^-18λ^6^-24λ^5^-35λ^4^-42λ^3^-26λ^2^-16λ-4

Scenario 2A: p(λ): -λ^8^+2λ^7^-18λ^6^+32λ^5^-39λ^4^+26λ^3^-14λ^2^+8λ+4

Scenario 2B: p(λ): -λ^8^-λ^7^-16λ^6^-13λ^5^-20λ^4^-23λ^3^-5λ^2^-5λ

**Model 3**

Baseline: p(λ): -λ^14^-2λ^13^-49λ^12^-77λ^11^-406λ^10^-587λ^9^-1227λ^8^-1469λ^7^-1493λ^6^-1098λ^5^-429λ^4^-6λ^3^+2λ^2^+56λ-4

Scenario 3A: p(λ): -λ^14^+2λ^13^-49λ^12^+95λ^11^-408λ^10^+531λ^9^-1091λ^8^+843λ^7^-967λ^6^+554λ^5^-55λ^4^+138λ^3^+30λ^2^-14λ+6

Scenario 3B: p(λ): -λ^14^+λ^13^-46λ^12^+44λ^11^-306λ^10^+170λ^9^-533λ^8^+89λ^7^-96λ^6^+15λ^5^+176λ^4^-36λ^3^+48λ^2^+91λ-32

Scenario 3C: p(λ): -λ^14^+5λ^13^-58λ^12^+228λ^11^-750λ^10^+1636λ^9^-2869λ^8^+3715λ^7^-3772λ^6^+3043λ^5^-1680λ^4^+528λ^3^+34λ^2^-133λ+56

**Model 4**

Baseline: p(λ): -λ^15^-λ^14^-54λ^13^-4λ^12^-401λ^11^+80λ^10^-732λ^9^+587λ^8^+513λ^7^+1349λ^6^+1915λ^5^+587λ^4^-33λ^3^-96λ^2^-115λ-6

Scenario 4A: p(λ): -λ^15^+3λ^14^-58λ^13^+196λ^12^-707λ^11^+1652λ^10^-3080λ^9^+4683λ^8^-4975λ^7^+4923λ^6^-2673λ^5^+1275λ^4^-1163λ^3^+194λ^2^-83λ+12

Scenario 4B: p(λ): -λ^15^+6λ^14^-70λ^13^+358λ^12^-1328λ^11^+3716λ^10^-7985λ^9^+13723λ^8^-18284λ^7^+19507λ^6^-15770λ^5^+9229λ^4^-4536λ^3^+1823λ^2^-632λ+220

Scenario 3C: p(λ): -λ^15^+2λ^14^-54λ^13^+134λ^12^-488λ^11^+984λ^10^-1397λ^9^+2105λ^8^-986λ^7^+991λ^6^+782λ^5^-1279λ^4^+326λ^3^-83λ^2^-132

Scenario 4D: p(λ): -λ^15^+4λ^14^-60λ^13^+244λ^12^-842λ^11^+2056λ^10^-3957λ^9^+6043λ^8^-6980λ^7^+6491λ^6^-3986λ^5^+1727λ^4^-1506λ^3^+1375λ^2^-984λ+356

Scenario 4E: p(λ): -λ^15^-52λ^13^+44λ^12^-362λ^11^+324λ^10^-721λ^9^+701λ^8^-94λ^7^+279λ^6^+774λ^5^-633λ^4^-460λ^3^+321λ^2^-20λ-228

**Model 5**

Baseline: p(λ): -λ^19^-2λ^18^-70λ^17^-87λ^16^-893λ^15^-1123λ^14^-4907λ^13^-6620λ^12^-15107λ^11^-20203λ^10^-28601λ^9^-32921λ^8^-31871λ^7^-25464λ^6^-15886λ^5^-4709λ^4^-500λ^3^+283λ^2^+223λ+33

Scenario 5A: p(λ): -λ^19^-2λ^18^-71λ^17^-87λ^16^-924λ^15^-1083λ^14^-4995λ^13^-5826λ^12^-14028λ^11^-1529λ^10^-21360λ^9^-20004λ^8^-16374λ^7^-9933λ^6^-3463λ^5^+986λ^4^-68λ^3^+292λ^2^-43λ-15

Scenario 5B: p(λ): -λ^19^-2λ^18^-72λ^17^-90λ^16^-969λ^15^-1190λ^14^-5497λ^13^-6743λ^12^-1634λ^11^-18297λ^10^-25415λ^9^-23030λ^8^-17090λ^7^-8171λ^6^-463λ^5^+3228λ^4^+733λ^3^+608λ^2^-67λ-20

Scenario 5C: p(λ): -λ^19^-2λ^18^-73λ^17^-91λ^16^-978λ^15^-1236λ^14^-5268λ^13^-7032λ^12^-14599λ^11^-18337λ^10^-22482λ^9^-22779λ^7^-18527λ^7^-11841λ^6^-7008λ^5^-2067λ^4^-823λ^3^-71λ^2^-34λ+3

Scenario 5D: p(λ): -λ^19^-2λ^18^-72λ^17^-89λ^16^-984λ^15^-1198λ^14^-5727λ^13^-6912λ^12^-17186λ^11^-18485λ^10^-25225λ^9^-20688λ^8^-12735λ^7^-2810λ^6^+4465λ^5^+6614λ^4^+2383λ^3^+1193λ^2^+11λ-2

Scenario 5E: p(λ): -λ^19^-2λ^18^-71λ^17^-90λ^16^-943λ^15^-1229λ^14^-5193λ^13^-6823λ^12^-14448λ^11^-16611λ^10^-20016λ^9^-16907λ^8^-9248λ^7^-1601λ^6^+3699λ^5^+4868λ^4^+1845λ^3^+667λ^2^+91λ+16

Scenario 5F: p(λ): -λ^19^-2λ^18^-71λ^17^-89λ^16^-956λ^15^-1226λ^14^-5322λ^13^-6669λ^12^-14223λ^11^-14334λ^10^-15229λ^9^-7544λ^8^+3541λ^7^+12036λ^6^+15044λ^5^+11981λ^4^+5032λ^3^+1700λ^2^+265λ+52
